# Coordination of Carbon and Nitrogen Metabolism Through Well-Timed Mid-Stage Nitrogen Compensation in Japonica Super Rice

**DOI:** 10.3390/plants13233351

**Published:** 2024-11-29

**Authors:** Qun Hu, Kaiwei Zhang, Weiqin Jiang, Shi Qiu, Guangyan Li, Fangfu Xu, Ying Zhu, Guodong Liu, Hui Gao, Hongcheng Zhang, Haiyan Wei

**Affiliations:** 1 Jiangsu Key Laboratory of Crop Cultivation and Physiology, Research Institute of Rice Industrial Engineering Technology, Jiangsu Co-Innovation Center for Modern Production Technology of Grain Crops, Yangzhou University, Yangzhou 225009, China; huqun@yzu.edu.cn (Q.H.); z15731020133@163.com (K.Z.); 008009@yzu.edu.cn (G.L.); xufangfu@yzu.edu.cn (F.X.); 008396@yzu.edu.cn (Y.Z.); guodongliu@yzu.edu.cn (G.L.); gaohui@yzu.edu.cn (H.G.); 2 Huaiyin Institute of Agricultural Sciences of Xuhuai Region in Jiangsu, Huai’an 223001, China; jwqdyx123@163.com; 3 Institute of Germplasm Resources and Biotechnology, Jiangsu Academy of Agricultural Sciences, Nanjing 210014, China; 20190054@jaas.ac.cn

**Keywords:** compensation timing, nitrogen uptake, soluble sugar, starch concentration, non-structural carbohydrates, yield, Japonica rice

## Abstract

The carbon and nitrogen (N) metabolism of rice under different mid-stage N compensation timings is unclear. Two Japonica super rice cultivars were examined under four N compensation timings (N1-N3: N compensation at mid-tillering, panicle initiation, and spikelet differentiation. N0: no N compensation) and CK with no N application. Mid-stage N compensation increased the N concentrations of various tissues, and N2 showed the highest plant N uptake at both the heading stage, maturity, and the grain filling period. Among the treatments, N2 showed the highest N utilization efficiency. With delayed compensation timing, there was a gradual decrease in soluble sugar and starch concentrations in each tissue, accompanied by a decline in the non-structural carbohydrate (NSC) concentration. Specifically, N2 treatment exhibited the highest NSC accumulation and the remobilized NSC reserve, but NSCs per spikelet decreased with delayed compensation timing. The highest yield was also obtained with N2, exhibiting a 4.5% increase compared to the N0 treatment, primarily due to an improvement in spikelets per panicle. Conclusively, N compensation at the panicle initiation stage is a reasonable N management strategy that can coordinate the improvement of carbon and N metabolism, enhance N accumulation with efficient utilization and NSC accumulation, and ultimately increase the yield.

## 1. Introduction

It is projected that approximately 670 million individuals will persistently experience hunger in 6 years, accounting for 8% of the global population. Rice (*Oryza sativa* L.) is globally recognized as a vital staple food crop, catering to the dietary needs of more than half of the global population [1]. Since the 1980s, rice production growth has decelerated; however, it is imperative to achieve a 50% to 70% increase in crop yields by 2050 in order to sustain the nutritional needs of nearly 10 billion people [2]. To accomplish this essential enhancement in production, substantial efforts should be focused on the development of novel rice varieties with high yield potential and the optimization of crop management practices [3,4]. The term “Super rice” refers to those varieties that exhibit significant advantages in key traits, such as high yield, superior grain quality, and strong resistance. The Super Rice Breeding Project was officially sanctioned by the Ministry of Agriculture of China in 1996, and the naming of super rice varieties commenced from the year 2006 [5]. As of 2024, the Ministry of Agriculture has designated a total of 129 super rice varieties, out of which 16% exhibit high-yield and high-quality traits of inbred Japonica super rice.

An additional crucial approach for optimizing nitrogen (N) fertilizer application involves addressing the environmental impacts associated with rice production [6]. N plays a pivotal role as the most crucial nutrient influencing crop productivity within intensive agricultural systems and serves as a key determinant in augmenting rice yield per unit area [7,8,9]. The mid-growth stage of rice is crucial for panicle differentiation and development, which coincides with the plant’s peak N demand [10]. Previous studies have demonstrated that the application of compensatory N at the initiation of panicle differentiation significantly enhances both the grain number per panicle and spikelet density per unit area. Conversely, supplying N during the spikelet differentiation phase effectively mitigates spikelet degeneration while maintaining photosynthetic activity and post-anthesis greenness [11,12,13,14]. While previous studies have extensively examined the impact of N application strategies on rice yields from a “source-sink” perspective, there has been limited studies into these phenomena through the lens of carbon–nitrogen metabolism.

N metabolism and carbon metabolism are the two most crucial metabolic processes in rice, characterized by their continuous coupling and mutual regulation [15]. N metabolism supplies enzymes and photosynthetic pigments crucial for carbon metabolism, while carbon metabolism provides the necessary carbon skeletons and assimilation capacity for N utilization [16]. The equilibrium and coordination between these two metabolic pathways, namely the absorption, utilization, and transport of N elements in conjunction with the synchronization of carbon fixation and carbohydrate production through photosynthesis, play a crucial role in facilitating rice growth, development, and yield formation [2,17]. Revisions in carbon metabolism are invariably accompanied by concomitant alterations in N metabolism. Therefore, the application of N fertilizer can effectively regulate both the directionality and proportionality of material fluxes within these pathways, thereby facilitating a synergistic accumulation of nitrogenous compounds alongside carbohydrates to optimize rice yields [18]. Previous studies suggested that the use of ultra-high yield cultivation management measures (N 270 kg ha^−1^) could increase the yield by 30% compared with the practical use of farmers (N 210 kg ha^−1^), and its flag leaves had a higher leaf photosynthetic rate, N and carbon metabolic enzyme activity, higher protective enzyme activity, and delayed leaf senescence [19]. Furthermore, it was found that, under aerobic irrigation, the best N fertilizer coupling scheme could improve rice yield and N utilization rate, which is due to the improvement of the photosynthetic rate in flag leaves and the activities of key enzymes of N and carbon metabolism, such as ribulose 1,5-diphosphate carboxylase/oxygenase and glutamine synthetase, and the increase in photosynthetic products and the total accumulation of N [20].

Previous studies on rice have predominantly focused on investigating the impact of N fertilizer application rates and different N application ratios on the enzymatic activity associated with carbon and N metabolism. Several studies have exclusively focused on the accumulation and transport of non-structural carbohydrates (NSCs) or the utilization and accumulation of N. However, there is a lack of research on the influence of mid-stage N compensation timing on N accumulation, NSC dynamics, transport mechanisms, and overall alterations in carbon-N metabolism and their subsequent impact on yield, particularly for self-pollinated Japonica super varieties with a high yield potential. Consequently, this study employs two extensively cultivated Japonica super rice varieties from the Yangtze River Delta region to investigate their responses to alterations in carbon and N metabolism induced by varying timings of nitrogen compensation. This investigation aims to elucidate the underlying mechanisms by which these self-pollinated Japonica super varieties adapt to fluctuations in N metabolism, thereby providing valuable theoretical insights for optimizing high-yielding and efficient cultivation practices for Japonica super rice.

## 2. Results

### 2.1. Tissue N Concentration at the Heading Stage and Maturity

Mid-stage N compensation timing had a significant influence on tissue N concentration at the heading stage and maturity. There were significant differences only in the stem N concentration between NG9108 and NG5055. Only the leaf and stem N concentration at the heading stage were significantly affected by the year (Table 1). Compared with the CK and N0 treatments, following mid-stage N compensation, the N concentrations of various tissues increased during both periods. Specifically, the leaf N concentration gradually increased with delayed N compensation timing, and the N3 treatment exhibited the highest N concentration, surpassing other treatments from 4.9% to 17.7% (at heading stage) and 14.4% to 52.3% (at maturity). The stem N concentration at the heading stage showed a consistent trend with leaf growth, but there were no significant differences in maturity among mid-stage N compensation treatments, except for 2018 in NG9108. The spike N concentration at the two stages did not differ significantly among mid-stage N compensation treatments, but it showed an overall increasing trend and was significantly higher than the CK. Comparing the two varieties, it was found that the stem N concentration at the two stages of NG5055 was higher than that of NG9108.

### 2.2. N Uptake and N Utilization Efficiency

As shown in Table 2, the analysis of variance shows that mid-stage N compensation treatment has significant effects on the N uptake of Japonica super rice. Compared to the N0 treatment, the N compensation treatments strongly increased plant N uptake at the heading stage (TN_HD_) and maturity (TN_MA_), with an average increase in the ranges of 18.5–31.7% and 19.1–32.9% for cultivar NG9108 and 17.8–31.4% and 19.0–34.8% for cultivar NG5055, respectively. TN_HD_ and TN_MA_ also showed a parabolic trend among N compensation treatments, reaching the maximum level during treatment N2, which also had a relatively high plant N uptake in the grain filling period (TN_HD-MA_).

**Figure 1:**
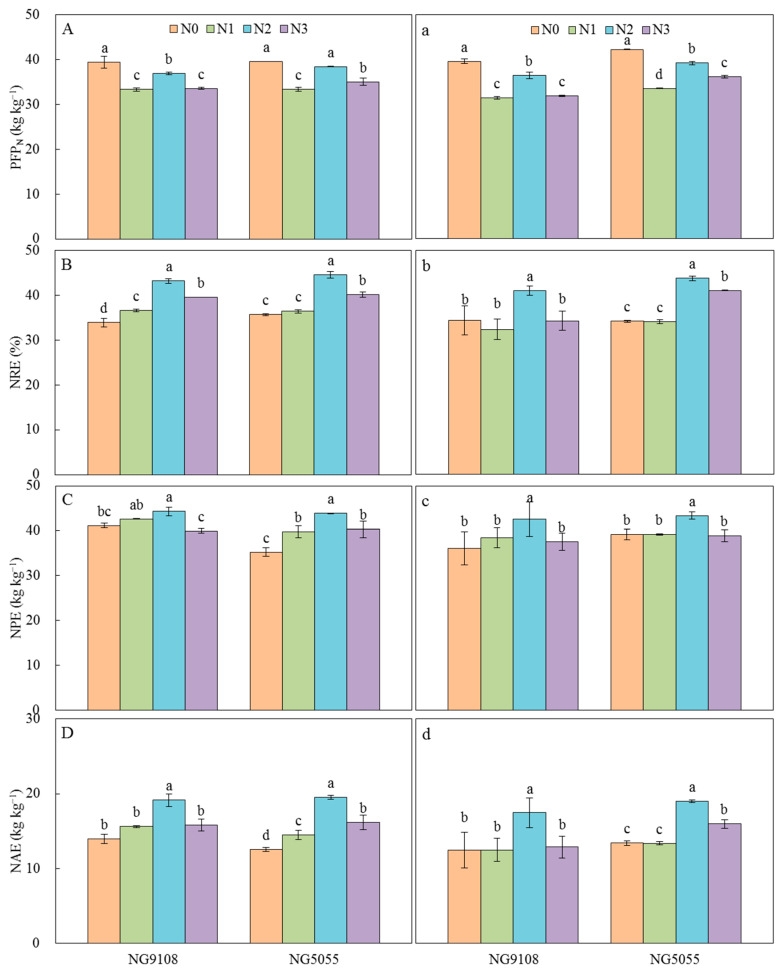
Effect of mid-stage nitrogen compensation timing on N utilization efficiency of Japonica super rice. PFP_N_, NRE, NPE, and NAE are the partial factor productivity of N fertilizer, N apparent recovery efficiency, N physiological use efficiency, and N agronomic use efficiency, respectively. Different letters indicate statistical significance at the 0.05 probability level. Error bars indicate the standard error. (**A**–**D**) represent the year 2017, and (**a**–**d**) represent the year 2018.

Compared with CK, the N utilization efficiency for grain production (NUEg), biomass production (NUEb), and N harvest index (NHI) decreased significantly after mid-stage N compensation. With the postponement of compensation timing, NUEg increased first and then decreased, and reached the maximum value in the N2 treatment, but NUEb and NHI showed a gradually decreasing trend. N apparent recovery efficiency (NRE), N physiological use efficiency (NPE), and N agronomic use efficiency (NAE) increased firstly and then decreased with the postponement of compensation timing, and N2 treatment had the highest value, which significantly increased by 4.9–17.7%, 14.4–52.3%, and 14.4–52.3% compared with other treatments, respectively (Figure 1). The partial factor productivity of N fertilizer (PFP_N_) during N0 treatment was the highest, followed by N2 treatment, and the difference between treatments was significant.

### 2.3. Concentration of Soluble Sugar and Starch

The analysis of variance in Table 3 and Table 4 reveals that, except for the spike soluble sugar concentration (SSconc) at the heading stage, which exhibits no significant differences across years, all other indicators of SSconc are significantly influenced by the year, variety, and mid-stage N compensation treatment. There was no significant difference in SSconc for all tissues at the heading stage compared with maturity, except for a slight decrease in the leaf number. The influence trend of mid-stage N compensation on the SSconc of each tissue was basically the same, which showed that it gradually decreased with the delay of the compensation timing. In contrast, there is considerable variation in the starch concentration (Sconc) among different tissues during the heading stage and maturity. The stem Sconc at maturity exhibited a significant decrease compared to that observed at the heading stage, whereas both leaf and spike Sconc demonstrated a significant increase relative to the heading stage. The variation trend of Sconc among all tissues in the two periods under mid-stage N compensation treatments was basically the same as that of soluble sugar. Specifically, spike Sconc for both varieties under N2 treatment at maturity are reduced by 8.0% and 9.4%, when compared to N0 treatment.

### 2.4. Dynamics of Tissue NSC Concentration After Anthesis

**Figure 2:**
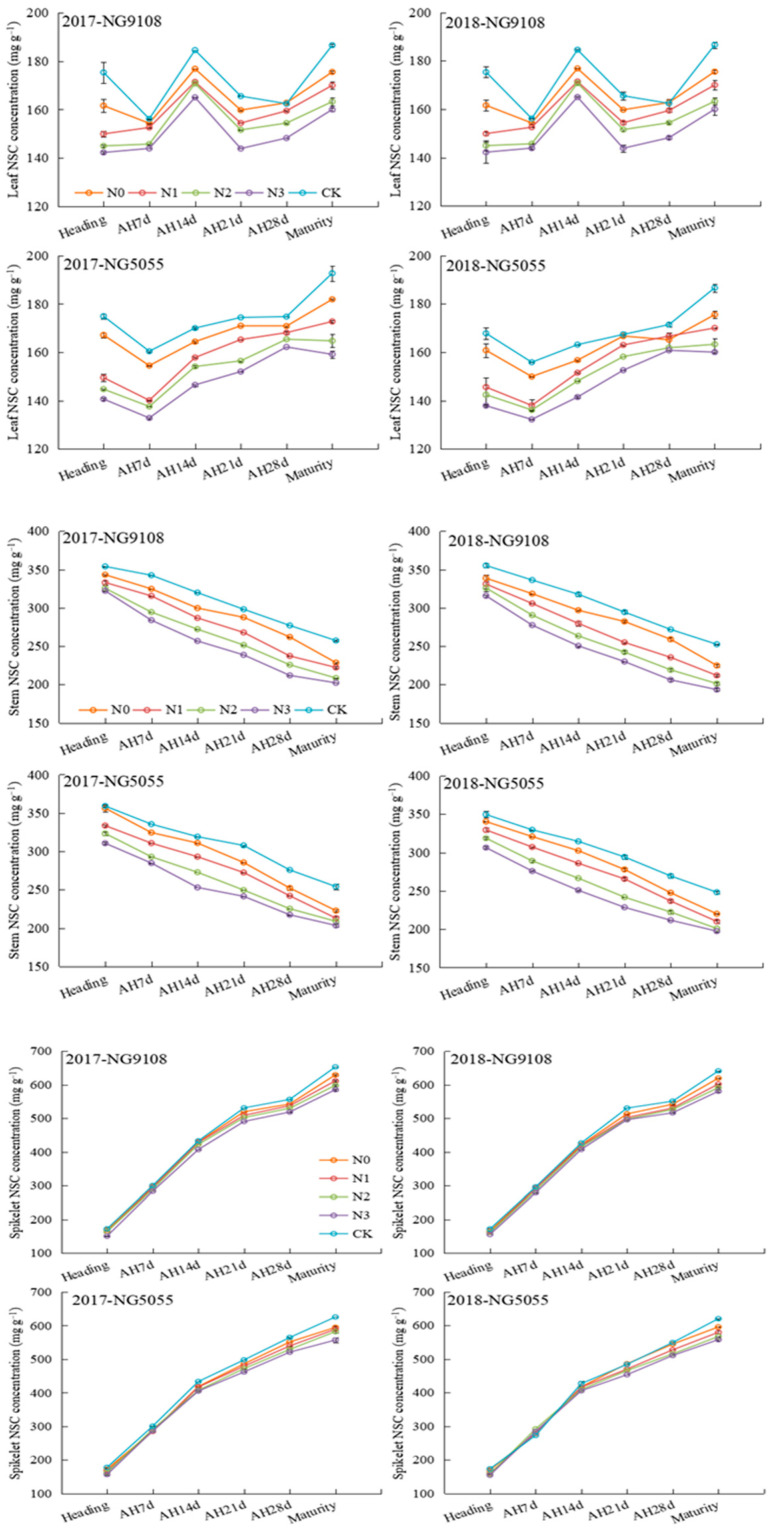
Effect of mid-stage nitrogen compensation timing on tissue NSC concentration of Japonica super rice.

The leaf NSC concentration remains relatively stable throughout the grain filling period, showing minimal variation between the heading stage and maturity, as depicted in Figure 2. However, it is significantly influenced by mid-stage N compensation treatment, which exhibited that there is a decline in leaf NSC concentration during grain filling as the timing of mid-stage nitrogen compensation is delayed. The stem NSC concentration gradually decreased over the same time, reaching the nadir at maturity, a trend which closely resembles that observed for leaf NSC concentration. Moreover, the spike NSC showed a consistent upward trajectory for both varieties during grain filling. The increasing trend was obvious in the first 14 days after heading, and the difference was small among different mid-stage N compensation treatments. From 14 days later, after heading, the difference of the spike NSC concentration among different treatments began to increase, and it gradually decreased with the delay of N compensation. The stem NSC concentration of the CK treatment was the highest at maturity, followed by N0 treatment, and N3 treatment was the lowest, in which NG9108 and NG5055 decreased by 9.4% and 10.1% compared with N0 treatment, respectively.

### 2.5. NSC Accumulation and Remobilization

Table 5 illustrates that mid-stage N compensation treatment significantly influences the accumulation and translocation of NSCs. Only the stem NSC contribution to grain is markedly affected by year, while NSC accumulation and remobilized NSC reserves at the heading stage and maturity exhibit a significant variation among different varieties. The trend of stem NSC accumulation during the two periods are consistent across both varieties, which initially increase before subsequently decreasing with the delay of N compensation timing. Specifically, N2 treatment had the highest NSC accumulation, and CK treatment had the least NSC accumulation in both periods, which was significantly lower than other treatments. NSC per spikelet showed a decline with the delayed compensation timing. Additionally, stem NSC translocation first increased and then decreased with the delay of compensation timing, and reached the maximum value following N2 treatment, which was significantly higher than other treatments. The stem NSC translocation of NG9108 and NG5055 varieties under N2 treatment were 2.49 times and 2.27 times the minimum value of CK treatment, respectively. Moreover, the remobilized NSC reserves of both N2 and N3 treatments are significantly higher than those of other treatments, and the highest value of two varieties under N2 treatment showed an increase by 34.3% and 24.6%, respectively, when compared to N0 treatment. Conversely, the stem NSC contribution to grain progressively declines with delayed N compensation timing across all treatments, where CK exhibits the lowest value.

### 2.6. Grain Yield and Yield Components

The mid-stage N compensation markedly enhanced the grain yield, exhibiting a parabolic trend across treatments, with the highest yield observed for the N2 treatment. NG9108 and NG5055 produced 10.0 and 10.4 t ha^−1^ in 2017 and 9.9 and 10.6 t ha^−1^ in 2018, respectively. The yield of N2 treatment for both NG9108 and NG5055 resulted in increases of 4.4% and 4.8% over N0 treatment in 2017, as well as increases of 4.3% and 4.5% in comparison to the N0 treatment in 2018, respectively. Compared with CK, mid-stage N compensation significantly enhanced the total spikelets per hectare. With the delay of N compensation, the total spikelets of the two varieties increased first and then decreased. Additionally, the panicle number decreased progressively with delayed compensation across both varieties, peaking following N1 treatment. The spikelets per panicle also reflected similar trends to those observed for yield and total spikelets, initially increasing then reaching the peak of N2 treatment and subsequently declining again. The grain filling percentage and 1000-grain weight increased with the delay of N compensation, and the treatment with late compensation was significantly higher than that with early compensation.

## 3. Discussion

### 3.1. Effects of Mid-Stage N Compensation Timing on Carbon and N Metabolism of Japonica Super Rice

N is a critical nutrient throughout the growth cycle of rice, and the effective absorption, accumulation, and utilization of N are key indicators for achieving a high yield and productive efficiency of rice cultivation [21,22,23]. This study reveals that mid-stage N compensation markedly improves N accumulation and plant N content at both the heading stage and maturity, compared to the N0 treatment, while also enhancing N uptake during later growth phases (Table 1 and Table 2), which align with previous research conclusions [13,24,25]. Prior research has indicated that the optimal timing for N compensation differs among various panicle types of rice varieties. Specifically, for rice with small panicles, nitrogen should be applied during the panicle differentiation stage. In contrast, rice with large panicles benefits from application either during the early spikelet differentiation stage or the heading stage [13]. Conversely, rice with middle-sized panicle exhibits minimal variation in response to nitrogen application across these three stages. In our study, two rice varieties with small panicles were examined and the postponement of mid-stage N compensation led to an initial increase, followed by a subsequent decline, in N accumulation at both the heading stage and maturity (Table 2). Therefore, N2 treatment exhibited peak values for compensatory N during the early panicle differentiation stage and demonstrated the highest NRE, NPE, and NAE values (Figure 1). These findings are consistent with prior research and suggest that N compensation at this stage may optimize N uptake and utilization characteristics in the two Japonica super rice varieties under investigation. N compensation timing here was mainly determined according to the nodes of the key growth period and leaf age model of the rice. The N concentration in each tissue at both the heading stage and maturity increased with the delay of the N compensation timing, while the soluble sugar and starch concentration correspondingly decreased, which reflects the inhibitory effect of N metabolism on carbon metabolism, and it is consistent with the previous research results [14,18]. A plausible explanation for this phenomenon is that a higher proportion of N-enriched photosynthates is utilized to provide carbon skeletons and isogeneity for N metabolism, thereby exerting a negative effect on carbon metabolism [26]. Consequently, both the activity and mRNA expression levels of starch branching enzymes in rice plants are relatively diminished, leading to a deceleration in starch synthesis, thus reducing the NSC content [27]. However, this decrease in NSC content was caused by the high level of carbon and N metabolism and the formation of a large population.

It was found that the accumulation and transport of NSCs in rice stems could be promoted by the right amount of mid-stage N supplementation, which was beneficial to grain filling, and the effects of different mid-stage N compensation timings on the accumulation and transport of NSCs in plants were different [28,29]. In this study, N2 treatment had the highest NSC accumulation per spikelet and stem at the heading stage and maturity (Table 5), while the N concentration, soluble sugar, and starch concentration across all tissue remained at moderate levels (Table 1, Table 3 and Table 4). The findings suggest that N2 treatment exerts a relatively minor inhibitory effect on plant N metabolism in relation to carbon metabolism, indicating a higher degree of coordination between carbon and N metabolism. Delayed N compensation increases the N content of rice in the later stages, promoting the conversion of NSCs into structural carbohydrates, such as those required for stem formation and root growth. This coordinated synergy facilitates the generation of increased levels of NSCs within the population, which can subsequently be transported to grains for accumulation. This study also showed that different N compensation timings influence assimilated distributions among various tissues, and N2 treatment had the largest NSC transfer volume and transport rate in the stem (Table 5), indicating that N compensation at the early panicle differentiation stage was conducive to mobilizing more assimilated substances stored in the stem sheath before flowering to be transported to the grains after flowering. Previous studies on large-panicle hybrid indica rice found that N compensation at the emergence of the top-third leaf was more effective for enhancing stem NSC accumulation and transport with yield improvement compared to N compensation at the emergence of the top-second leaf and top-fourth leaf [30]. The observed discrepancy may indicate inherent variations in growth characteristics among different types and varieties of rice, supporting our investigation into the diversity of rice cultivars. It is noteworthy that, during the grain filling process, the stem NSC concentrations of N2 and N3 treatments decreased more rapidly at 0–14 days than 14 days later after heading, while the decrease rate was slower in the first 14 days after heading under N1 treatment (Figure 2). The observed phenomenon can be attributed to the accelerated rates of NSC accumulation from late panicle differentiation to the heading stage under N2 and N3 treatments, leading to a substantial storage of NSCs in the stem that are rapidly transferred to the grains during the early filling phase [31]. Leaf photosynthetic performance remained high due to the sufficient N supply during the middle and late stages of filling, enhancing its contribution to grain filling processes. Consequently, the contribution rate of NSC transport from the stem to grains decreased compared with both N0 and N1 treatments (Table 2 and Table 5).

### 3.2. Effects of Carbon and N Metabolism on the Yield of Japonica Super Rice

During the vegetative growth stage of rice, N compensation initially enhances the plant’s N metabolism, facilitating the synthesis of chlorophyll and associated enzymes crucial for carbon metabolism [26]. This process promotes the production of photosynthetic products, primarily channeled into structural carbohydrates essential for tissue development, with a portion being stored in the stem as soluble sugars, such as starch and sucrose [32,33]. The middle growth stage of rice refers to the late tillering stage to the heading and flowering stage of rice, which corresponds to the transition from vegetative growth to reproductive growth and is characterized by a rapid establishment of all organs, particularly the stem and panicle, accompanied by an increased N demand in plants [34]. In this study, compared to the N0 treatment, N compensation during this critical period led to a significantly higher number of effective panicles, spikelets per panicle, total spikelets, and overall yield (Table 6). These findings align with the previous research [13,28,30], indicating that mid-stage N compensation is conducive to coordinating the improvement of carbon and N levels, promoting the simultaneous growth and development of vegetative organs and reproductive organs, and ultimately forming a large “source sink” [35]. Notably, among the treatments assessed, N2 exhibited the highest total spikelets while N1 and N3 showed comparable spikelets and yield (Table 6). The analysis of yield structure showed that N compensation during the panicle initiation stage could synchronize the panicle number and grains per panicle to coordinate the increase in population total spikelets. Additionally, N compensation during mid-tillering had a greater impact on panicle number formation, while during the spikelet differentiation stage it had a certain effect on grains per panicle. Previous researchers believed that for medium- and small-panicle Japonica super rice varieties, a sufficient panicle number is a necessary prerequisite for a high yield, and on this basis, it is necessary to coordinate grains per panicle to form a larger population of spikelets [36].

After heading, the plant enters the reproductive growth period, which mainly requires grain filling, namely carbon metabolism, as the main task, and is the final and decisive stage of yield. The accumulation of NSCs in the panicles was partly due to the transport of NSCs stored in the stem before flowering, and the most important part was due to the photosynthesis of leaves after flowering [37]. N2 treatment enhances carbon–nitrogen metabolic activity pre-anthesis, promoting increased NSC accumulation in the stem and establishing a foundational biomass basis for a higher yield potential (Table 6). Some studies have suggested that the transfer volume of NSCs after heading is closely related to the number of spikelets, and the more spikelets there are, the more NSCs will be transferred from the stem during grain filling, which may explain the reason for the high transfer volume and transfer rate of N2 treatment NSCs in this study. In addition, N2 treatment promoted N uptake after anthesis due to delayed N compensation, retained the functional period of leaves for a longer time, and increased the contribution of post-anthesis photosynthetic production to grain NSCs (Table 2). Considering these factors collectively suggests that an optimal mid-stage N compensation should be during the panicle initiation stage for these two super rice varieties. At this stage, elevated levels of carbon-N metabolism facilitate abundant and evenly distributed photosynthetic products that are preferentially converted into essential structural carbohydrates required for tissue development. This process results in a significant accumulation of NSCs in the stem at the heading stage while enabling the leaves to sustain prolonged periods of efficient NSC production post-flowering [38].

## 4. Materials and Methods

### 4.1. Site Description

**Figure 3:**
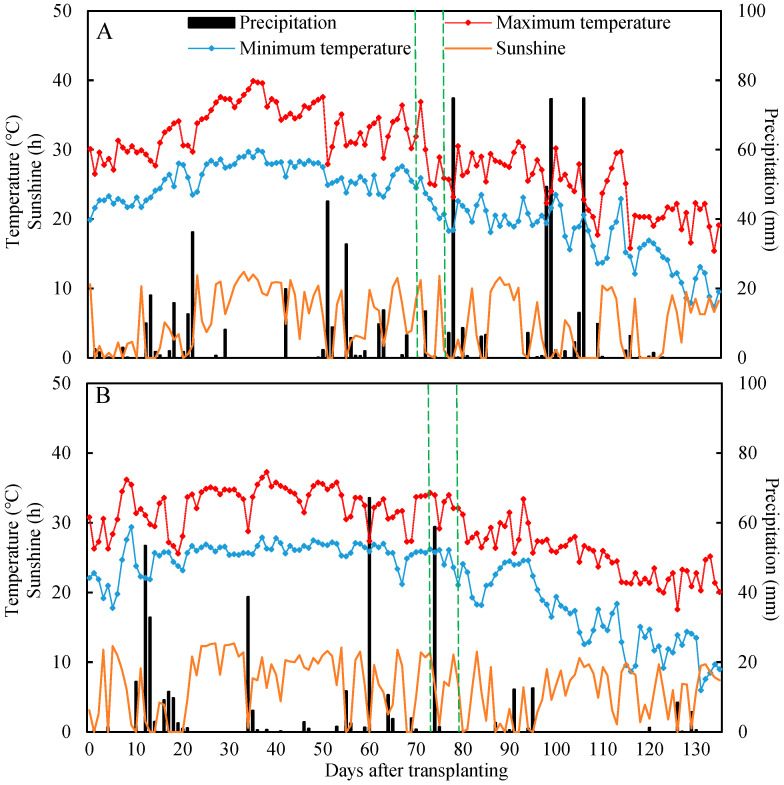
Daily temperature, sunshine duration, and precipitation during rice growing season in 2017 (**A**) and 2018 (**B**) at a field site in Yazhou Town, Jiangsu Province, China. Dotted lines in green indicate the heading times for Nangeng 9108 and Nangeng 5055.

Field experiments were conducted during rice growing seasons (May–October) in 2017 and 2018 in Yazhou Town, Hai’an City, Jiangsu Province, China (32°43′ N, 120°32′ E, 5 m altitude). The study area was situated in the north, subtropical, humid climatic zone, characterized by an average annual temperature of 14.6 °C, mean annual precipitation of 1021.9 mm, and an average sunshine duration of 2052.6 h. The previous crop in the experimental field was wheat (the yield was about 6.7 t hm^−2^). The field soil was high sandy soil with 21.6 g kg^−1^ organic matter, 1.4 g kg^−1^ total N, 89.4 mg kg^−1^ alkaline hydrolyzed N, 16.8 mg kg^−1^ available *p*, and 78 mg kg^−1^ available K. The average daily temperature, sunshine hours, and precipitation during the rice growing seasons in 2017 and 2018 were collected from a weather station situated close to the experimental field, and these data can be found in Figure 3.

### 4.2. Experimental Design and Crop Management

Two Japonica super rice cultivars “Nangeng 9108” (NG9108) and “Nangeng 5055” (NG5055), which are widely cultivated in the lower reaches of the Yangtze River, were used as the food plant material in this experiment. The super rice certification for NG9108 was obtained in 2015, while NG5055 received its certification in 2014. Their seeds were sown in special plastic nursery trays outdoors on 15 May 2017 and 2018, and their resultant seedlings were transplanted into the field on 18 June 2017 and 2018. Hill spacing was fixed at 30 cm × 12.4 cm, with four seedlings planted per hill (107.52 × 10^4^ seedlings per hectare).

The experiment was arranged in a split-plot design, with the two rice cultivars as the main plot factor and four timings of mid-stage N compensation as the within-plot factor. Treatment design and mid-stage N compensation timing are shown in Table 7. Briefly, four applications were employed: no N compensation (N0), one-time N compensation at the mid-tillering stage (N1), panicle initiation stage (N2), and spikelet differentiation stage (N3). There was another treatment without N compensation as a control (CK). The plot area was 18 m^2^ (6 m × 3 m). The within-plot area was repeated three times as a unit, and there were 30 plots in total. Total N applied was equivalent to 270 kg ha^−1^ for three N compensation treatments and 189 kg ha^−1^ for N0, of which 40% of N was applied in the transplanting period, 30% used as tillering N 7 days after transplanting, and a final 30% applied as compensated N. The source of N applied was urea. Other practices implemented in the experiment conformed to the local recommendations from government agencies.

### 4.3. Field Sampling and Lab Analyses

#### 4.3.1. N Content

At the heading stage and maturity, three typical hills of rice were selected from each plot based on the average stem and tiller count per hill. In the laboratory, these were segmented into stems, leaves, and panicles (only stems and leaves at the jointing stage). Samples underwent enzyme inactivation at 105 °C for 30 min and were dried at 80 °C until reaching a constant weight. Post-weighing, the samples were digested using H_2_SO_4_-H_2_O_2_, and the N content was ascertained through the semimicro Kjeldahl method, followed by the calculation method of total N accumulation at the heading stage (TN_HD_) and maturity (TN_MA_), total N accumulation from heading to maturity (TN_HD-MA_), nitrogen use efficiency for grain production (NUEg), nitrogen use efficiency for biomass production (NUEb), nitrogen harvest index (NHI), partial factor productivity of fertilize N (PFP_N_), N apparent recovery efficiency (NRE), N physiological use efficiency (NPE), and N agronomic use efficiency (NAE).

TN_HD_ = Above-ground dry matter accumulation at heading stage × Plant N content (1)

TN_MA_ = Above-ground dry matter accumulation at maturity × Plant N content (2)

TN_HD-MA_ = TN_MA_ − TN_HD_ NUEg = Grain yield/TN_MA_ (3)

NUEb = Above-ground dry matter accumulation at maturity/TN_MA_ (4)

NHI = Grain N accumulation at maturity/TN_MA_ PFP_N_ = Grain yield/Total N application rate (5)

NRE = (total N uptake in N supply − total N uptake in CK)/N supply rate(6)

NAE = (grain yield in N supply-grain yield in CK)/N supply rate(7)

NPE = NAE/NRE(8)

#### 4.3.2. Soluble Sugar and Starch Content

Accurately measure 0.1 g of the dry sample and transfer it into a 10 mL centrifuge tube for extraction, followed by the addition of 8 mL of 80% ethanol. The supernatant was extracted using a water bath and centrifuge, and the soluble sugar extract was obtained through three repeated extractions. Extract 1 mL of soluble sugar using a pipette gun and transfer it into a heat-resistant test tube with a capacity of 10 mL. Gradually add anthracone reagent to the test tube, and determine the absorbance value at a wavelength of 620 nm using an enzyme label.

The formula for calculating SSconc is as follows:

SSconc (%) = [(C × V/a)/(W × 1000)] × 100 (9)

where C = Glucose value obtained from the standard curve (mg); V = Total volume of sample extract (mL); a = Volume of extraction solvent collected during color development (mL); W = Sample weight (g).

Add 8 mL of 80% ethanol to the residue obtained after extracting soluble sugar. After subjecting it to a water bath and centrifugation, dry and cool the residue at 80 °C. Subsequently, we boiled the residue in distilled water for 2 h and then performed extractions it with 9.2 mol L^−1^ of perchloric acid 3 times. The resulting supernatant was then transferred to the same volumetric bottle and diluted with distilled water to a final volume of 100 mL in order to obtain the starch extract. Starch concentration (Sconc) is determined by the same method as SSconc.

The formula for calculating Ssconc is as follows:

Sconc (%) = [(C × V/a) × 0.9/(W × 1000)] × 100(10)

where C = Glucose value obtained from the standard curve (mg); V = Total volume of sample extract (mL); a = Volume of extraction solvent collected during color development (mL); W = Sample weight (g); 0.9 = Conversion coefficient of glucose to starch.

#### 4.3.3. Rice Yield and Its Components

All rice plants from 100 holes in the middle of each plot were hand harvested at maturity, and the grain yield was weighed. The final grain yield was adjusted to a 14% moisture content. The number of panicles per m^2^ was determined from three representatives square meter regions that were randomly sampled from each plot. The number of spikelets per panicle, percentage of filled grains, and filled grain weight were determined from plants from five representative hills. The percentage of filled grains was determined as the ratio of filled grains to the total number of spikelets.

### 4.4. Statistical Analysis

Analysis of variance (ANOVA) was performed using SPSS 20.0 software for Win. Means were tested by the least significant difference at *p* = 0.05 (LSD0.05). Graphs were prepared by Excel 2013 for Win, and all error bars indicated standard errors of means.

## 5. Conclusions

Different N compensation timings had significant effects on the carbon and N metabolism of Japonica super rice. N compensation at the panicle initiation stage could increase the N accumulation after jointing and total N uptake at maturity, and improve the N use efficiency. In terms of carbon metabolism, N compensation at the panicle initiation stage could enhance NSC accumulation in the stems during later growth stages, as well as facilitate stem NSC translocation and remobilized NSC reserves after heading. Therefore, this study concludes that, for inbred Japonica super rice NG9108 and NG5055, N compensation at the panicle initiation stage is a reasonable N management strategy that can coordinate the improvement of carbon and N metabolism, promote the establishment of high yields, enhance N accumulation with efficient utilization and promote the formation of NSCs, and ultimately increase the yield.

## Figures and Tables

**Table 1:** Effect of mid-stage nitrogen compensation timing on tissue N concentration at heading stage and maturity of Japonica super rice.

Year	Cultivar	Treatment	Heading Stage			Maturity		
			Nconc Leaf (mg N g^−1^)	Nconc Stem (mg N g^−1^)	Nconc Spike (mg N g^−1^)	Nconc Leaf (mg N g^−1^)	Nconc Stem (mg N g^−1^)	Nconc Spike (mg N g^−1^)
2017	NG9108	N0	10.75 b	19.60 bc	11.20 a	5.25 c	12.81 b	11.57 b
		N1	10.98 b	19.08 c	10.74 ab	5.74 b	14.02 a	11.51 b
		N2	11.04 b	19.97 b	11.02 ab	5.81 b	13.77 a	11.62 ab
		N3	11.85 a	21.72 a	11.48 a	6.73 a	14.11 a	12.11 a
		CK	10.64 b	17.88 d	10.00 b	4.84 d	10.70 c	11.36 b
	NG5055	N0	11.00 bc	19.90 b	11.24 ab	5.45 b	13.02 c	11.57 ab
		N1	11.06 bc	20.01 b	10.78 b	5.33 b	14.01 b	12.12 a
		N2	11.23 b	20.17 b	11.66 a	5.74 b	14.45 ab	11.73 ab
		N3	12.00 a	22.47 a	11.58 a	6.76 a	14.67 a	12.17 a
		CK	10.64 c	18.02 c	9.99 c	4.44 c	11.92 d	11.02 b
2018	NG9108	N0	10.68 c	19.05 b	10.52 b	5.43 d	13.07 d	11.50 b
		N1	10.92 bc	19.76 b	11.39 a	5.62 c	13.39 c	11.89 a
		N2	11.06 b	19.52 b	11.41 a	5.84 b	13.97 b	12.01 a
		N3	11.60 a	20.75 a	11.92 a	6.68 a	14.53 a	12.16 a
		CK	10.23 d	15.42 c	9.72 c	4.44 e	10.73 e	10.90 c
	NG5055	N0	10.56 c	19.38 b	10.77 b	5.45 b	12.81 bc	11.51 b
		N1	10.92 b	19.50 b	11.20 ab	5.52 b	13.60 ab	11.94 ab
		N2	11.08 b	19.87 b	11.37 a	5.72 b	13.96 a	11.72 ab
		N3	12.17 a	21.75 a	11.78 a	6.82 a	14.61 a	12.11 a
		CK	10.34 c	17.72 c	9.72 c	4.94 c	12.08 c	10.63 c
F value	Year (Y)		**	**	NS	NS	NS	NS
	Cultivar (C)		NS	**	NS	NS	**	NS
	Treatment (T)		**	**	**	**	**	**
	Y × C		NS	NS	NS	*	NS	NS
	Y × T		NS	*	**	NS	NS	*
	C × T		NS	NS	NS	NS	**	*
	Y × C × T		NS	**	NS	*	NS	NS

Values with a column followed by different letters are significantly different at *p* < 0.05. * Significant at *p* < 0.05; ** significant at *p* < 0.01.

**Table 2:** Effect of mid-stage nitrogen compensation timing on N uptake and N utilization efficiency of Japonica super rice.

Year	Cultivar	Treatment	TN_HD_ (kg ha^−1^)	TN_HD-MA_ (kg ha^−1^)	TN_MA_ (kg ha^−1^)	NUEg (kg kg^−1^)	NUEb (kg kg^−1^)	NHI (%)
2017	NG9108	N0	126.35 d	20.35 c	146.70 d	50.70 b	103.49 b	69.54 b
		N1	152.70 c	28.71 b	181.41 c	49.66 bc	99.51 b	70.33 ab
		N2	167.14 a	32.12 a	199.26 a	50.01 bc	98.80 b	68.44 c
		N3	157.66 b	31.70 a	189.36 b	47.89 c	92.91 c	67.54 d
		CK	70.86 e	11.74 d	82.59 e	58.12 a	109.14 a	70.78 a
	NG5055	N0	130.01 d	21.90 c	151.92 d	49.22 c	101.72 b	66.64 b
		N1	156.25 c	26.66 b	182.90 c	49.30 c	99.09 bc	66.43 b
		N2	171.93 a	32.92 a	204.85 a	50.67 b	97.92 c	66.31 b
		N3	161.24 b	31.63 a	192.87 b	49.10 c	92.73 d	65.59 b
		CK	74.00 e	10.50 d	84.50 e	60.38 a	110.93 a	71.98 a
2018	NG9108	N0	126.62 c	25.28 c	151.90 c	49.28 b	101.00 b	69.20 c
		N1	146.94 b	27.49 bc	174.42 b	48.74 b	98.45 b	71.40 b
		N2	165.93 a	31.72 a	197.65 a	49.82 b	98.71 b	68.72 c
		N3	149.88 b	29.66 ab	179.54 b	47.95 b	93.92 c	67.01 d
		CK	71.89 d	15.15 d	87.05 d	58.95 a	111.57 a	74.41 a
	NG5055	N0	133.03 d	21.72 c	154.75 d	51.60 b	104.44 b	67.32 bc
		N1	153.50 c	28.56 b	182.06 c	49.76 cd	99.61 c	67.01 c
		N2	173.77 a	34.64 a	208.41 a	50.78 bc	98.48 c	68.39 ab
		N3	167.32 b	33.59 a	200.91 b	48.59 d	92.88 d	66.89 c
		CK	78.53 e	11.65 d	90.17 e	60.52 a	111.41 a	69.39 a
F value	Year (Y)		NS	**	NS	NS	NS	**
	Cultivar (C)		**	NS	**	**	NS	**
	Treatment (T)		**	**	**	**	**	**
	Y × C		**	NS	**	NS	NS	NS
	Y × T		**	**	**	NS	NS	NS
	C × T		**	**	**	NS	NS	**
	Y × C × T		**	**	**	*	NS	**

Values with a column followed by different letters are significantly different at *p* < 0.05. * Significant at *p* < 0.05; ** significant at *p* < 0.01. TN_HD_, TN_HD-MA_, TN_MA_, NUEg, NUEb, and NHI are the total nitrogen accumulation at the heading stage, total nitrogen accumulation from heading to maturity, total nitrogen accumulation at maturity, nitrogen use efficiency for grain production, nitrogen use efficiency for biomass production, and nitrogen harvest index, respectively.

**Table 3:** Effect of mid-stage nitrogen compensation timing on tissue soluble sugar concentration at the heading stage and maturity of Japonica super rice.

Year	Cultivar	Treatment	Heading Stage			Maturity		
			SSconc Leaf (mg g^−1^)	SSconc Stem (mg g^−1^)	SSconc Spike (mg g^−1^)	SSconc Leaf (mg g^−1^)	SSconc Stem (mg g^−1^)	SSconc Spike (mg g^−1^)
2017	NG9108	N0	102.58 b	108.96 b	100.39 a	101.24 b	113.69 a	101.50 b
		N1	100.41 b	105.69 b	98.99 b	94.91 c	110.17 b	97.96 c
		N2	96.34 c	101.56 c	98.62 b	93.12 d	100.74 c	95.52 d
		N3	95.24 c	99.95 c	92.73 c	91.13 e	100.24 c	88.86 e
		CK	118.45 a	114.27 a	100.64 a	102.63 a	115.42 a	106.19 a
	NG5055	N0	104.56 b	114.38 a	99.52 ab	98.74 b	110.38 b	95.54 b
		N1	96.81 c	109.12 b	96.22 bc	95.04 c	102.71 c	94.09 b
		N2	95.19 c	103.62 c	94.97 c	91.42 d	99.69 d	93.38 b
		N3	92.07 d	98.32 d	92.89 c	87.62 e	95.13 e	89.28 c
		CK	108.60 a	115.10 a	102.25 a	103.10 a	119.05 a	104.12 a
2018	NG9108	N0	101.78 b	106.30 b	98.91 ab	97.79 a	110.71 a	98.14 b
		N1	96.09 d	103.00 bc	98.75 ab	93.54 b	103.62 b	95.53 c
		N2	94.10 d	101.12 c	98.11 bc	91.64 bc	96.50 c	91.82 d
		N3	93.66 d	96.40 d	96.30 c	89.54 c	92.18 d	85.48 e
		CK	109.13 a	114.00 a	100.92 a	100.06 a	112.87 a	102.60 a
	NG5055	N0	100.34 b	111.77 a	99.30 a	95.14 b	105.03 b	94.47 b
		N1	95.17 c	107.78 b	94.14 b	93.24 bc	101.31 c	92.64 b
		N2	93.67 cd	101.74 c	94.84 b	89.91 cd	95.81 d	90.96 bc
		N3	90.61 d	97.24 d	93.72 b	87.49 d	93.27 e	87.10 c
		CK	105.20 a	112.39 a	100.63 a	99.20 a	112.78 a	100.36 a
F value	Year (Y)		**	**	NS	**	**	**
	Cultivar (C)		**	**	**	**	**	**
	Treatment (T)		**	**	**	**	**	**
	Y × C		NS	NS	NS	NS	*	NS
	Y × T		*	NS	*	NS	NS	NS
	C × T		**	*	**	NS	**	**
	Y × C × T		*	NS	NS	NS	**	NS

Values with a column followed by different letters are significantly different at *p* < 0.05. * Significant at *p* < 0.05; ** significant at *p* < 0.01. SSconc: soluble sugar concentration.

**Table 4:** Effect of mid-stage nitrogen compensation timing on tissue starch concentration at the heading stage and maturity of Japonica super rice.

Year	Cultivar	Treatment	Heading Stage			Maturity		
			Sconc Leaf (mg g^−1^)	Sconc Stem (mg g^−1^)	Sconc Spike (mg g^−1^)	Sconc Leaf (mg g^−1^)	Sconc Stem (mg g^−1^)	Sconc Spike (mg g^−1^)
2017	NG9108	N0	60.54 b	234.15 b	68.80 b	81.62 b	114.89 b	528.11 b
		N1	58.22 bc	227.35 c	64.71 c	79.35 c	112.09 c	513.90 c
		N2	53.33 cd	223.97 d	65.15 c	76.01 d	107.73 d	503.13 d
		N3	52.08 d	222.28 d	57.78 d	74.22 e	101.98 e	496.74 e
		CK	74.45 a	239.70 a	71.39 a	90.21 a	141.94 a	546.99 a
	NG5055	N0	62.61 b	241.90 a	73.14 ab	83.19 b	125.01 b	499.07 b
		N1	52.71 c	224.46 b	70.34 bc	77.72 bc	110.16 c	494.89 bc
		N2	49.55 d	219.74 c	67.67 c	73.34 cd	109.08 c	488.76 c
		N3	48.56 e	212.24 d	63.78 d	71.54 d	104.88 d	467.73 d
		CK	66.33 a	243.97 a	75.78 a	89.63 a	139.54 a	521.50 a
2018	NG9108	N0	59.85 b	232.37 b	66.62 b	77.76 b	114.17 b	521.47 b
		N1	53.84 c	227.99 bc	63.34 c	76.49 c	108.28 c	506.69 c
		N2	50.85 cd	224.78 c	63.45 c	71.64 d	104.78 d	499.73 d
		N3	48.58 d	219.31 d	59.30 d	70.49 e	101.28 e	495.29 e
		CK	66.26 a	241.21 a	70.75 a	86.57 a	139.63 a	538.59 a
	NG5055	N0	60.46 a	228.49 b	70.10 b	80.35 b	114.99 b	500.75 b
		N1	50.40 b	221.59 c	66.05 c	74.66 c	108.81 c	486.75 c
		N2	48.77 b	216.88 c	62.85 d	71.68 d	105.08 cd	475.84 d
		N3	47.26 b	209.06 d	60.52 e	69.93 d	104.16 d	470.47 d
		CK	62.62 a	237.07 a	72.29 a	84.70 a	135.38 a	519.51 a
F value	Year (Y)		**	**	**	**	**	**
	Cultivar (C)		**	**	**	NS	*	**
	Treatment (T)		**	**	**	**	**	**
	Y × C		NS	**	**	NS	*	NS
	Y × T		*	**	NS	NS	NS	*
	C × T		**	**	*	*	**	*
	Y × C × T		NS	**	NS	NS	**	*

Values with a column followed by different letters are significantly different at *p* < 0.05. * Significant at *p* < 0.05; ** significant at *p* < 0.01. Sconc: starch concentration.

**Table 5:** Effect of mid-stage nitrogen compensation timing on stem NSC accumulation and remobilization of Japonica super rice.

Year	Cultivar	Treatment	NSC Per Spikelet (mg)	NSC at Heading (g m^−2^)	NSC at Maturity (g m^−2^)	NSC Translocation (g m^−2^)	Remobilized NSC Reserve (%)	Stem NSC Contribution to Grain (%)
2017	NG9108	N0	6.19 a	188.6 b	111.1 c	77.5 c	41.1 b	10.4 a
		N1	5.41 c	210.0 a	117.6 b	92.3 ab	43.6 b	10.1 ab
		N2	5.20 cd	218.4 a	121.6 a	96.8 a	44.3 a	9.7 ab
		N3	4.94 d	188.2 b	103.3 d	84.9 bc	45.1 a	9.4 bc
		CK	5.74 b	116.7 c	75.6 e	41.1 d	35.2 c	8.6 c
	NG5055	N0	6.30 a	192.5 c	113.6 b	78.9 c	41.0 b	10.5 a
		N1	5.86 b	218.4 b	127.3 a	91.0 b	41.7 ab	10.1 b
		N2	5.19 c	224.1 a	128.1 a	96.0 a	42.8 a	9.3 c
		N3	4.83 d	189.4 d	109.9 c	79.4 c	41.9 ab	8.4 d
		CK	5.68 b	118.3 e	76.8 d	41.5 d	35.1 c	8.1 d
2018	NG9108	N0	6.41 a	192.4 c	111.3 b	81.1 bc	42.2 c	10.8 a
		N1	5.63 b	201.5 b	114.5 ab	87.1 b	43.2 bc	10.2 b
		N2	5.17 c	213.4 a	118.1 a	95.3 a	44.7 a	9.7 c
		N3	5.05 c	181.3 d	101.8 c	79.5 c	43.9 ab	9.2 c
		CK	5.54 b	114.9 e	76.7 d	38.2 d	33.3 d	7.4 d
	NG5055	N0	6.26 a	195.5 c	121.0 a	74.5 c	38.1 c	9.3 a
		N1	5.44 b	208.7 b	123.6 a	85.1 b	40.8 b	9.4 a
		N2	5.10 c	221.7 a	123.8 a	98.0 a	44.2 a	9.3 a
		N3	5.02 c	197.1 c	111.2 b	85.9 b	43.6 a	8.8 b
		CK	5.51 b	121.6 d	78.5 c	43.1 d	35.5 d	7.9 c
F value	Year (Y)		NS	NS	NS	NS	NS	**
	Cultivar (C)		NS	**	**	NS	**	**
	Treatment (T)		**	**	**	**	**	**
	Y × C		**	*	*	NS	NS	NS
	Y × T		**	**	**	NS	NS	**
	C × T		NS	NS	**	NS	**	*
	Y × C × T		**	*	*	**	**	**

Values with a column followed by different letters are significantly different at *p* < 0.05. * Significant at *p* < 0.05; ** significant at *p* < 0.01.

**Table 6:** Effect of mid-stage nitrogen compensation timing on grain yield and yield components of Japonica super rice.

Year	Cultivar	Treatment	Grain Yield (t ha^−1^)	Panicles (per m^2^)	Spikelets (per Panicle)	Total Spikelets (10^3^ per m^2^)	Grain Filling Percentage (%)	Grain Weight (mg)
2017	NG9108	N0	7.44 c	310.1 c	98.3 d	30.5 c	93.2 ab	27.0 b
		N1	9.01 b	335.6 a	115.0 c	38.6 b	90.8 c	26.9 b
		N2	9.96 a	323.5 b	129.7 a	42.0 a	92.3 b	27.1 ab
		N3	9.07 b	312.8 c	121.8 b	38.1 b	93.2 ab	27.2 ab
		CK	4.80 d	224.2 d	90.7 e	20.3 d	94.4 a	27.7 a
	NG5055	N0	7.48 d	323.5 c	94.5 d	30.6 d	94.5 b	26.9 b
		N1	9.02 c	358.4 a	104.0 c	37.3 c	92.3 d	26.9 b
		N2	10.38 a	342.3 b	126.1 a	43.2 a	93.5 c	26.9 b
		N3	9.47 b	334.3 bc	117.3 b	39.2 b	93.9 c	27.1 b
		CK	5.10 e	241.7 d	86.2 e	20.8 e	95.2 a	27.6 a
2018	NG9108	N0	7.48 c	327.6 c	91.6 d	30.0 c	95.8 ab	26.7 b
		N1	8.50 b	353.1 a	101.4 c	35.8 b	93.9 c	26.7 b
		N2	9.85 a	346.4 b	119.3 a	41.3 a	94.7 bc	26.5 b
		N3	8.61 b	327.6 c	109.6 b	35.9 b	95.2 ab	27.1 ab
		CK	5.13 d	228.2 d	90.9 d	20.7 d	95.8 a	27.8 a
	NG5055	N0	7.99 d	332.9 c	93.8 d	31.2 d	95.3 a	27.8 ab
		N1	9.06 c	363.8 a	105.5 c	38.4 c	94.0 c	26.6 c
		N2	10.58 a	353.1 b	123.1 a	43.4 a	94.6 b	26.9 c
		N3	9.76 b	347.7 b	112.9 b	39.3 b	94.9 b	27.4 b
		CK	5.46 e	244.3 d	90.4 e	22.1 e	95.6 a	28.1 a
F value	Year (Y)		NS	**	**	NS	**	NS
	Cultivar (C)		**	**	**	**	*	*
	Treatment (T)		**	**	**	**	**	**
	Y × C		**	**	**	**	**	**
	Y × T		**	**	**	*	NS	**
	C × T		**	**	NS	*	NS	NS
	Y × C × T		NS	NS	*	NS	NS	NS

Values with a column followed by different letters are significantly different at *p* < 0.05. * Significant at *p* < 0.05; ** significant at *p* < 0.01.

**Table 7:** Nitrogen treatment under different mid-stage nitrogen compensation timings (kg N ha^−1^).

Treatment	Basal Nitrogen (1 Day Before Transplanting)	Tillering Nitrogen (7 Days After Transplanting)	Mid-Stage Nitrogen Compensation Timing			Total Nitrogen
			Mid-Tillering (43–45 Days Before Heading)	Panicle Initiation (33–35 Days Before Heading)	Spikelet Differentiation (18–20 Days Before Heading)	
N0	94.5	94.5	0	0	0	189
N1	94.5	94.5	81	0	0	270
N2	94.5	94.5	0	81	0	270
N3	94.5	94.5	0	0	81	270
CK	0	0	0	0	0	0

## Data Availability

The data are contained within the article.
